# Elevated basal luteinizing hormone does not impair the outcome of human menopausal gonadotropin and medroxyprogesterone acetate treatment cycles

**DOI:** 10.1038/s41598-018-32128-4

**Published:** 2018-09-14

**Authors:** Lihua Sun, Jing Ye, Yun Wang, Qiuju Chen, Renfei Cai, Yonglun Fu, Hui Tian, Qifeng Lyu, Xuefeng Lu, Yanping Kuang

**Affiliations:** 1 Department of Assisted Reproduction, Shanghai Ninth People’s Hospital, Shanghai Jiaotong University School of Medicine, 639 Zhizaoju Rd, Shanghai, 200011 China; 2 Department of Assisted Reproduction, Shanghai East Hospital, Shanghai Tongji University School of Medicine, Shanghai, 200120 China

## Abstract

The potential effects of high basal luteinizing hormone (LH) levels on human reproduction were controversial. To demonstrate the effects of elevated basal LH levels on the outcome of patients undergoing *in vitro* fertilization/intracytoplasmic sperm injection (IVF/ICSI) cycles, we performed a retrospective data analysis of 1011 polycystic ovarian syndrome (PCOS) patients treated with human menopausal gonadotropin and medroxyprogesterone acetate (hMG + MPA) protocol at our center between Nov. 2013 and Jun. 2017. PCOS patients with elevated basal LH levels had significantly higher LH exposure during the stimulation period. The group with LH ≥ 10 mIU/mL showed a lower mean total hMG dose used but higher numbers of oocytes retrieved, metaphase II oocytes, embryos and top-quality embryos developed than the groups with lower basal LH levels. Moreover, partial correlation analysis showed that the basal LH level was negatively correlated with the total hMG dose but positively correlated with the numbers of oocytes retrieved, metaphase II oocytes, embryos, and top-quality embryos. There were no significant differences in the rates of oocyte retrieval, fertilization, implantation, clinical pregnancy and miscarriage between the groups based on frozen embryo transfer (FET). We concluded that elevated basal LH level does not impair the final outcome of hMG + MPA-treated IVF/ICSI cycles in PCOS women.

## Introduction

Luteinizing hormone (LH) plays an essential physiological role in follicle steroidogenesis and development and oocyte maturation. However, the optimal LH level during ovarian stimulation continues to be controversial. Additionally, it is still unknown whether individual patients with a high level of LH would actually benefit from presuppression of LH^1–3^. Polycystic ovary syndrome (PCOS) is often associated with high endogenous LH secretion^4^. High basal LH levels have been associated with significant decreases in oocyte maturation and fertilization rates and impaired embryo quality, consequently resulting in an impaired pregnancy rate and higher miscarriage rate^5,6^. High exposure of the genital tract to LH in the early follicular phase was found to be associated with a reduced chance of pregnancy in cycles involving stimulation with recombinant FSH and a GnRH antagonist for *in vitro* fertilization /intracytoplasmic sperm injection (IVF/ICSI)^7^. On the other hand, in PCOS patients undergoing IVF, the continuous suppression of endogenous gonadotropins for the initiation of stimulation might be associated with a slower pattern of follicular development and lower E2 levels. Inclusion of exogenous LH in the ovarian stimulation protocol can have beneficial or detrimental effects on oocyte yield and quality^8^. A recent study reported a higher incidence of top-quality preimplantation embryos when adding LH activity to FSH stimulation in women undergoing IVF^9,10^. Moreover, stimulation protocols that include LH may decrease the euploidy rates of preimplantation embryos^11^. The contradictory findings regarding LH levels for ovarian stimulation protocols indicate that LH had adverse effects on the fertility process.

Recently, we found that medroxyprogesterone acetate (MPA) can be safely used to prevent premature LH surges in women undergoing controlled ovarian stimulation (COS) for IVF^12^. Compared with a short agonist protocol, this protocol is more flexible and has also been applied among patients with PCOS or without PCOS, with no difference in the data for stimulation, number of oocytes collected and ongoing pregnancy rates^12–14^. Moreover, this protocol has an advantage in that GnRHa could be used to trigger reductions in exogenous hCG stimulation and OHSS incidence (unpublished data). The mode of LH suppression in hMG + MPA is different from that in GnRH agonist (GnRHa)-treated or GnRH antagonist-treated cycles. In GnRHa-treated cycles, GnRHa induces desensitization, which mostly results in high and stable LH levels during the entire stimulation period^15,16^. In GnRH antagonist-treated cycles, the LH levels rapidly decline after the start of the GnRH antagonist treatment^17^. In the hMG + MPA protocol, LH levels are unsuppressed at the initiation of stimulation and gradually decrease, which would allow higher LH levels during the stimulation period. However, the effect of an elevated basal LH level on the outcome of the IVF stimulation cycle using the hMG + MPA protocol is still unknown. This retrospective study was performed to answer these questions by analyzing a large PCOS population with IVF/ICSI cycles treated using the hMG + MPA protocol (n = 1011).

## Results

### Patient characteristics

**Figure 1:**
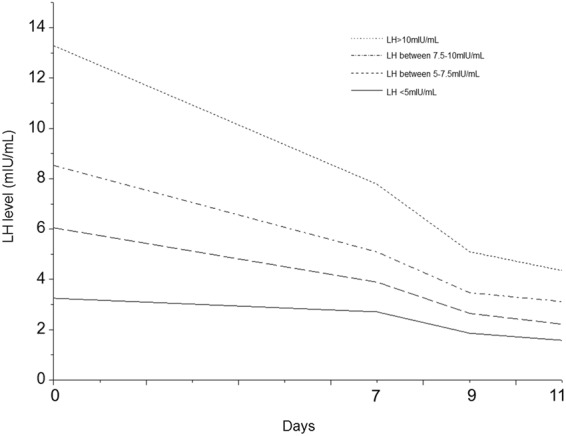
Median serum LH levels in the patients on stimulation days 0, 7, 9, and 11. The differences in serum LH among the four groups are significant on days 0, 7, 9, and 11 (P < 0.001). Group with LH < 5 mIU/mL: solid line; group with LH between 5 and 7.5 mIU/mL: dashed line; group with LH between 7.5 and 10 mIU/mL: dashed-dotted line; group with LH ≥ 10 mIU/mL: dotted line.

During the study period, 10.39% (105/1011) of the PCOS patients had an elevated basal LH level (LH ≥ 10 mIU/mL) after undergoing a COS cycle using the hMG + MPA protocol^18^. A gradual decrease in serum LH was observed after MPA administration, which indicated that there was a subgroup of patients exposed to high levels of LH during their early follicular development (Fig. 1). The effect of high basal LH on the outcome of hMG + MPA-treated IVF/ICSI cycles is still unknown. To evaluate the effect of high basal LH level on the outcome of the COS cycle, the cycles were divided into four groups according to their basal LH level: LH < 5 mIU/mL, LH between 5 and 7.5 mIU/mL (including 5), LH between 7.5 and 10 mIU/mL (including 7.5), and LH ≥ 10 mIU/mL. A comparison of the patient characteristics indicated that the average age of the patients with LH ≥ 10 mIU/mL was 30.77 years, which was significantly younger than the patients with basal LH values between 5 and 7.5 mIU/mL and those with basal LH values < 5 mIU/mL (30.77 ± 3.25 vs. 31.97 ± 3.57 and 30.77 ± 3.25 vs. 32.00 ± 3.69, respectively, P < 0.05) (Table 1). The BMI of patients with LH ≥ 10 mIU/mL was significantly lower than that of the patients with basal LH values between 5 and 7.5 mIU/mL, those with basal LH values between 7.5 and 10 mIU/mL, and those with basal LH values < 5 mIU/mL (22.12 ± 3.13 vs. 23.90 ± 12.24, 22.12 ± 3.13 vs. 23.41 ± 4.04, and 22.12 ± 3.13 vs. 23.98 ± 4.07, respectively, P < 0.05) (Table 1). This result is consistent with a previous study showing that circulating LH levels were inversely correlated with body weight^18^. Moreover, patients with LH ≥ 10 mIU/mL had an FSH level similar to the level of the patients with basal LH values between 7.5 and 10 mIU/mL and between 5 and 7.5 mIU/mL but had significantly higher basal FSH levels than the patients with basal LH < 5 mIU/mL (6.00 ± 1.30 vs. 4.88 ± 1.28, P < 0.05). However, no significant between-group differences existed in the basal E2 and P levels (Table 1).

**Table 1:** Baseline patient characteristics.

Characteristic	LH (mIU/mL)			
	<5 (n = 575)	5–7.5 (n = 216	7.5–10 (n = 115)	≥10 (n = 105)
Age of women	32.00 ± 3.69^a^	31.97 ± 3.57^a^	31.31 ± 3.14	30.77 ± 3.25
BMI of women	23.98 ± 4.07^a^	23.41 ± 4.04^a^	23.90 ± 12.24^a^	22.12 ± 3.13
Basal FSH (mIU/mL)	4.88 ± 1.28	5.73 ± 1.27^b^	5.67 ± 1.21^b^	6.00 ± 1.30^b^
Basal E2 (pg/mL)	32.95 ± 16.62	36.25 ± 14.65	41.59 ± 22.09	41.22 ± 20.00
Basal P (ng/mL)	0.27 ± 0.20	0.26 ± 0.18	0.28 ± 0.19	0.32 ± 0.24

Data are presented as the mean ± SD. BMI: body mass index. ^a^P < 0.05 vs. the group with LH ≥ 10 mIU/mL; ^b^P < 0.05 vs. the group with LH < 5 mIU/mL.

### Ovarian stimulation characteristics and outcomes

**Figure 2:**
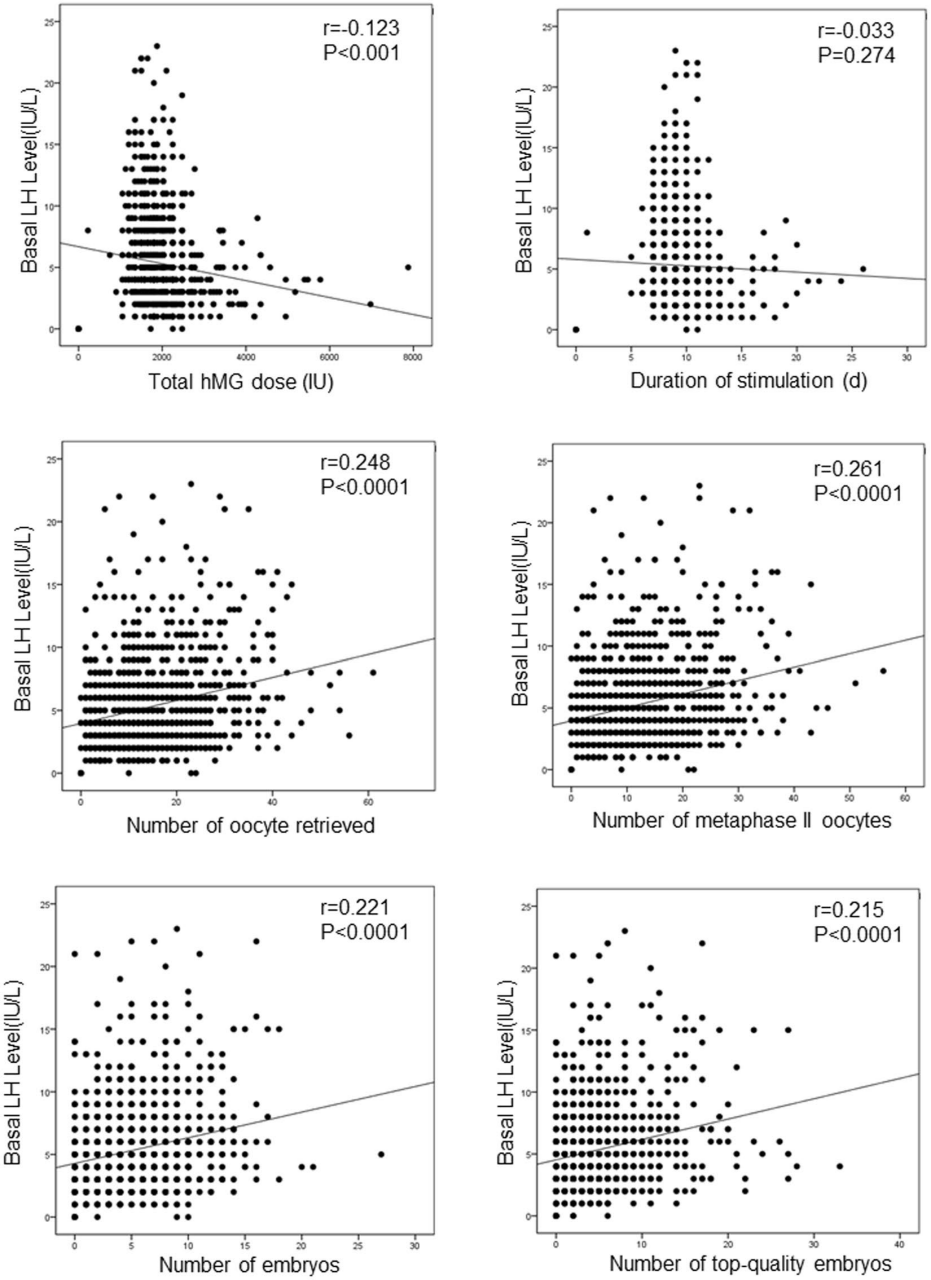
Correlation of basal LH level with total hMG used, days of ovarian stimulation, number of oocytes retrieved, number of metaphase II oocytes, number of embryos, and number of top-quality embryos.

The ovarian stimulation characteristics of patients with basal LH < 5 mIU/mL, basal LH between 5 and 7.5 mIU/mL, basal LH between 7.5 and 10 mIU/mL, and basal LH ≥ 10 mIU/mL are presented in Table 2. Exposure of the genital tract to LH was assessed by the area under the curve (AUC) calculated from the day of stimulation, day 0, to the day of trigger. A significantly higher exposure of the genital tract to LH was present in patients with basal LH ≥ 10 mIU/mL than in the groups with basal LH between 7.5 and 10 mIU/mL, basal LH between 5 and 7.5 mIU/mL, and basal LH < 5 mIU/mL (96.26 ± 30.31 vs. 57.54 ± 31.57, 96.26 ± 30.31 vs. 42.01 ± 13.83, and 96.26 ± 30.31 vs. 26.51 ± 10.79, P < 0.001 for all) (Table 2). The total hMG dose in the group with basal LH ≥ 10 mIU/mL was significantly less than that in the group with basal LH between 5 and 7.5 mIU/mL and that in the group with basal LH < 5 mIU/mL (1786.36 ± 362.79 vs. 1971.98 ± 627.15, 1786.36 ± 362.79 vs. 2096.16 ± 610.37, P < 0.05 for all). However, the numbers of oocytes aspirated, oocytes retrieved and metaphase II oocytes were significantly greater in the groups with basal LH ≥ 10 mIU/mL than the groups with basal LH between 7.5 and 10 mIU/mL, basal LH between 5 and 7.5 mIU/mL, and basal LH < 5 mIU/mL (P < 0.01 for all). Consistent with these results, the mean E2 level in the group with LH ≥ 10 mIU/mL on the trigger day was higher than the mean value in the group with basal LH between 5 and 7.5 mIU/mL and the group with basal LH < 5 mIU/mL (4478.49 ± 1163.26 vs. 3802.67 ± 1547.59 and 4478.49 ± 1163.26 vs. 3727.09 ± 1492.93, respectively, P < 0.001) (Table 2). These results are in agreement with the findings of a previous study showing that compared with hMG, treatment with pure FSH required significantly more ampules of gonadotropin but resulted in significantly fewer leading follicles and lower serum E2 concentrations^19^. The mean LH level on the trigger day in the groups with LH ≥ 10 mIU/mL was significantly higher than those in the group with basal LH between 7.5 and 10 mIU/mL, the group with basal LH between 5 and 7.5 mIU/mL, and the group with basal LH < 5 mIU/mL (4.15 ± 2.44 vs. 3.18 ± 2.20, 4.15 ± 2.44 vs. 2.45 ± 4.43, 4.15 ± 2.44 vs. 1.62 ± 1.78, respectively, P < 0.05) (Table 2). There were no significant differences in the mean FSH level on the trigger day among the groups with basal LH < 5 mIU/mL, basal LH between 5 and 7.5 mIU/mL, basal LH between 7.5 and 10 mIU/mL, and basal LH ≥ 10 mIU/mL. Our previous results showed that P elevation during hMG + MPA cycles was the result of ovarian stimulation and was driven by the high E2 levels and the number of oocytes. Consistent with these results, here, we found the P level on the trigger day in the groups with LH ≥ 10 mIU/mL were significantly higher than those in the group with basal LH between 5 and 7.5 mIU/mL, the group with basal LH between 7.5 and 10 mIU/mL, and the group with basal LH < 5 mIU/mL (1.14 ± 0.97 vs. 0.84 ± 0.75, 1.14 ± 0.97 vs. 0.66 ± 0.65, and 1.14 ± 0.97 vs. 0.56 ± 0.69, respectively, P < 0.05) (Table 2). There were more embryos and top-quality embryos that developed in the group with basal LH ≥ 10 mIU/mL than in the groups with basal LH between 7.5 and 10 mIU/mL, basal LH between 5 and 7.5 mIU/mL, and basal LH < 5 mIU/mL (P < 0.05 for all). However, there were no significant between-group differences in the ratio of frozen blastocysts to frozen embryos on day 3 (Table 2). Furthermore, partial correlation analysis showed the basal LH level was negatively correlated with the total hMG dose (r = 0.123, P < 0.001) but positively correlated with the number of oocytes retrieved (r = 0.248, P < 0.0001), number of metaphase II oocytes (r = 0.261, P < 0.0001), number of embryos (r = 0.221, P < 0.0001), and number of top-quality embryos (r = 0.215, P < 0.0001) when the influences of age, BMI and basal FSH level were excluded (Fig. 2). However, there were no significant between-group differences in oocyte retrieval rate and fertilization rate, indicating that the greater number of oocytes retrieved and embryos developed was due to a high ovarian response to hMG stimulation in the PCOS patients with an elevated LH level. There were no significant between-group differences in the implantation rate, clinical pregnancy rate, miscarriage rate per transfer, or ectopic pregnancy rate per transfer (Table 3). These results demonstrated that the elevated LH level in PCOS patients undergoing IVF treatment with the hMG + MPA protocol did not have a negative effect on the outcome of IVF/ICSI.

**Table 2:** Ovarian stimulation characteristics.

Characteristic	LH (mIU/mL)			
	<5 (n = 575)	5–7.5 (n = 216)	7.5–10 (n = 115)	≥10 (n = 105)
LH AUC	26.51 ± 10.7^a^	42.01 ± 13.8^a^	57.54 ± 31.5^a^	96.26 ± 30.3^a^
Total hMG dose (IU)	2096.16 ± 610.37^b^	1971.98 ± 627.15^b^	1880.23 ± 461.02	1786.36 ± 362.79
Duration of stimulation (d)	11.54 ± 2.15	11.26 ± 2.09^c^	11.11 ± 2.00	11.02 ± 1.31^c^
Number of oocytes aspirated	21.86 ± 13.27^d^	25.12 ± 15.50 ^d^	26.16 ± 15.3^d^	31.97 ± 15.52
Number of oocytes retrieved	13.38 ± 8.45^d^	16.23 ± 10.08^d^	16.32 ± 11.13^d^	19.71 ± 10.52
Number of metaphase II oocytes	11.10 ± 7.24^d^	13.97 ± 8.65^d^	13.47 ± 9.38^d^	17.18 ± 9.60
Oocyte retrieval rate	62.63 ± 21.69	65.74 ± 21.99	63.82 ± 23.23	63.13 ± 19.12
Normal fertilization rate	96.65 ± 43.08	95.75 ± 42.21	90.70 ± 36.27	90.92 ± 32.22
Number of embryos	4.66 ± 4.20^e^	5.52 ± 4.92^e^	5.30 ± 3.97^e^	6.83 ± 5.60
Top-quality embryos	4.62 ± 3.56^e^	5.46 ± 3.85^e^	5.26 ± 3.47^e^	6.82 ± 4.20
Ratio of blastocysts to day 3 frozen embryos	0.23 ± 0.61	0.27 ± 0.55	0.25 ± 0.51	0.30 ± 0.56
FSH (mIU/mL)	12.44 ± 4.76	13.28 ± 4.83	12.40 ± 5.29	13.05 ± 4.90
LH (mIU/mL)	1.62 ± 1.78 ^f^	2.45 ± 4.43^f^	3.18 ± 2.20^f^	4.15 ± 2.44^f^
E2 (pg/mL)	3727.09 ± 1492.93^b^	3802.67 ± 1547.59^b^	4284.46 ± 1238.60	4478.49 ± 1163.26
P (ng/mL)	0.56 ± 0.69^f^	0.66 ± 0.65^f^	0.84 ± 0.75^f^	1.14 ± 0.97^f^

Data are presented as the mean ± SD. ^a^P < 0.001; ^b^P < 0.001 vs. the group with LH ≥ 10 mIU/mL; ^c^P < 0.05 vs. the group with LH < 5 mIU/mL; ^d^P < 0.01 vs. the group with LH ≥ 10 mIU/mL; ^e^P < 0.05 vs. the group with LH ≥ 10 mIU/mL; ^f^P < 0.05. AUC: area under the curve.

**Table 3:** FET cycle outcomes.

Variable	LH (mIU/mL)				P Value
	<5 (n = 575)	5–7.5) (n = 216	7.5–10 (n = 115)	≥10 (n = 105)	
Implantation rate (%)	43.29 (481/1111)	43.48 (197/453)	49.32 (109/221)	44.03 (96/218)	NS
Clinical pregnancy rate per transfer (%)	47.68 (288/604)	46.47 (112/241)	58.82 (70/119)	55.5 (61/110)	NS
Miscarriage rate per transfer (%)	5.9 (36/604)	5.8 (14/115)	5.04 (6/119)	3.64 (4/110)	NS
Ectopic pregnancy rate per transfer (%)	0.50 (3/604)	0.41 (1/115)	0 (0/216)	0.91 (1/110)	NS

NS: not significant.

## Discussion

LH is a crucial physiological regulator of the human menstrual cycle. The effect of the basal LH level on reproductive function is still unclear. Compared to those in healthy women, the absolute level of circulating LH and its level relative to the FSH level are significantly higher in women with PCOS. LH activity had previously been deemed potentially detrimental to reproductive function. A previous report showed that IVF had a significantly reduced success rate in patients with an elevated basal serum LH level^20^. Moreover, high concentrations of LH during the follicular phase in women with polycystic ovaries have a deleterious effect on conception rate and may be a causal factor for early pregnancy loss^7^. For the IVF/ICSI cycles treated with the hMG + MPA protocol, the high LH level might be maintained in the early stimulation period in PCOS patients with a high basal LH level since the LH level slowly decreased after MPA administration was performed. Consistent with this proposal, we showed that PCOS patients with an elevated basal LH level were exposed to higher LH on stimulation days 7, 9, and 11 compared with that in the patients with a lower basal LH level during the follicular phase in hMG + MPA-treated IVF/ICSI cycles.

Our results showed no significant differences in the rates of implantation, clinical Npregnancy and miscarriage among the groups with different basal LH levels during the IVF cycles treated with the hMG + MPA protocol. Moreover, more embryos and more top-quality embryos developed in the group with LH > 10 mIU/ml than in the other groups. These results indicated that a high basal LH level did not have any negative effects on oocyte quality and embryo quality and did not lead to poor outcomes of IVF/ICSI treatment. These findings were different from the observed lower pregnancy rates in the group with higher LH levels in GnRH antagonist-treated cycles^21^. This difference might account for the changes in LH levels in the hMG + MPA protocol that were different from that in the GnRH antagonist-treated cycles, in which the LH levels decreased substantially after GnRH antagonist administration. However, in the hMG + MPA protocol-treated cycles, the LH level decreased gradually. We presumed that not only the LH level but also an inappropriate LH action were interfering with follicular and oocyte development. In addition, we noted that PCOS patients with an elevated basal LH level had a higher P level on the trigger day. This result was consistent with those of our previous study showing an elevated P level on the trigger day in the cycles with more oocyte development, which might be the result of ovarian stimulation and which was driven by the high E2 levels and number of oocytes^22^. Our previous report showed the elevated P on the trigger day had no negative effect on embryo quality based on FET. Thus, excluding the negative effect of an elevated P level on endometrial receptivity during FET might be another major reason for our result of no negative effect on the outcome of hMG + MPA-treated IVF cycles in PCOS patients with an elevated LH level.

Moreover, we noted that less hMG was needed for the patients with basal LH > 10 mIU/ml but more oocytes aspirated and oocytes retrieved were needed in the group with basal LH > 10 mIU/ml compared to the other groups. Furthermore, exNcluding the influences of age, BMI, and basal FSH level, the basal LH level was positively correlated with the number of oocytes retrieved. These results indicate that patients with an elevated basal LH level might have a higher response to hMG stimulation. These results are in agreement with those of previous reports showing that the addition of exogenous LH to the ovarian stimulation protocol can have beneficial effects on oocyte yield. However, whether the addition of exogenous LH to the hMG + MPA protocol has benefits for patients with a low basal LH level needs further study.

In conclusion, this is the first study demonstrating the dynamics of the LH level during IVF cycles in PCOS patients treated with the hMG + MPA protocol. Our results showed that the hMG + MPA protocol could be effectively used for PCOS patients with a high basal LH level, and there was little evidence supporting presuppression of LH before hMG + MPA stimulation in PCOS patients with an elevNated LH level. A gradual decrease in the LH level might lead to less interference with follicular and oocyte development.

## Methods

### Study population and design

This was a retrospective analysis of a cohort with the IVF/ICSI cycles (n = 1011) Nof 1011 PCOS patients, performed at the Department of Assisted Reproduction of the Ninth People’s Hospital of Shanghai Jiaotong University School of Medicine during the period from November 2013 to June 2017. PCOS was defined according to the Rotterdam ESHRE/ASRMS-sponsored PCOS Consensus Workshop Group as two of the following three criteria: (1) oligo- or anovulation, (2) clinical and/or biochemical signs of hyperandrogenism, and (3) polycystic ovaries^23^. This study was approved by the ethics committee (institutional review board) of the Ninth People’s Hospital of Shanghai. Informed consent for the study and for the release of patient records was obtained from the patients. The trial was conducted in accordance with the principles of the Declaration of Helsinki for medical research.

Patients underwent COS using hMG (150–225 IU/d; Anhui Fengyuan Pharmaceutical Co., Anhui, China) and MPA (10 mg/d), as previously described, from menstrual cycle (MC) day 3^12^. The final stage of oocyte maturation was triggered using triptorelin (0.1 mg; decapeptyl, Ferring Pharmaceuticals, Malmo, Sweden) cotriggered by SC (subcutaneous) injections of triptorelin (0.1 mg) and hCG (1,000–2000 IU; Lizhu Pharmaceutical Trading Co., Zhuhai, China). Transvaginal ultrasound-guided oocyte retrieval was conducted 34–36 hours after the trigger. All follicles with a diameter greater than 10 mm were retrieved. Fertilization of the aspirated oocyte was performed *in vitro* by either conventional insemination or ICSI depending on the semen parameters. Embryos were examined for the number and regularity of blastomeres and the degree of embryonic fragmentation on the third day according to the criteria of Cummins *et al*.^24^. All good-quality embryos (including grade 1 and grade 2 8-cell embryos) were frozen by vitrification on the third day after oocyte retrieval. Embryos that were not top-quality were placed in extended culture until they reached the blastocyst stage. However, if there were more than 8 top-quality embryos, only 8 top-quality embryos were frozen, and all other embryos continued in culture. During this stage, only blastocysts with good morphology were frozen on day 5 or 6. The vitrification procedure for freezing cleavage-stage embryos and blastocysts was previously described^12^. For thawing, solutions of 1, 0.5, and 0 M sucrose were used sequentially as cryoprotectant dilutions. All vitrification and warming steps were performed at room temperature except for the first warming step, which was conducted at 37 °C.

Embryo and endometrium synchronization with FET was performed using a previously described method^12,14,25^. Briefly, we used letrozole and, if necessary, hMG to stimulate monofollicular growth. The common method used was as follows: 5 mg of letrozole was administered from cycle day 3 to 5, and follicle growth was then monitored beginning on day 10. At specified times, the treatment included a low dose of hMG (75 IU/day) to stimulate follicular and endometrial lining growth. Administration of 5000 IU hCG and FET timing was performed as described elsewhere. The hormone-substituted cycle was performed for patients with thin endometria during the stimulation cycles using daily oral administration of 75 mg/day EE (Xinyi Pharmaceutical Co., Shanghai, China) from day 3 to meet the criterion of an endometrial thickness of 8 mm. At that time, patients were given 0.4 g of progestin (Utrogestan, Laboratoires Besins-Iscovesco, Paris, France) intravaginally and 8 mg estradiol valerate daily (Abbott Biological B.V., the Netherlands). The day 3 embryos were used for transfer first if there were both frozen day 3 embryos and blastocysts. The maximum number of transferred embryos was 2 per patient. The progesterone supplementation was continued until 10 weeks of gestation after pregnancy was achieved.

### Hormone Analysis

Serum FSH, LH, E2, and P levels were measured on MC3 (stimulation day 0), stimulation days 7 and 9, the trigger day, and the day after trigger (approximately 10 hours after the injection of GnRHa and/or hCG). The hormone levels were measured with chemiluminescence (Abbott Biologicals B.V., Weesp, Netherlands). The lower limits of sensitivity were as follows: FSH, 0.06 IU/L; LH, 0.09 IU/L; E2, 10 pg/mL; and P, 0.1 ng/mL. The upper limit of the E2 measurement was 5,000 pg/mL. If the E2 levels were higher than the upper limit, the E2 values were recorded as 5,000 pg/mL.

### Statistical Analysis

The primary outcome measure was the number of top-quality embryos. The secondary outcome measures included the FET implantation rate and clinical pregnancy rate. The number of good-quality embryos was the number of all good-quality embryos on day 3. Clinical pregnancy was defined as the presence of a gestational sac with fetal heart activity during ultrasound examination 7 weeks after FET. The implantation rate was defined as the number of gestational sacs divided by the number of transferred embryos. The data in the table are presented as the mean ± SD. Comparisons among groups were analyzed by ANOVA followed by an appropriate post hoc test. P < 0.05 was considered statistically significant. Pearson’s correlation coefficient (r) was calculated for correlation analysis. All data were analyzed using Statistical Package for the Social Sciences for Windows (ver. 22, SPSS Inc.). The levels of total exposure to LH were expressed as the area under the curve (AUC) from the day of stimulation, day 0, to the day of hCG administration.

## Data Availability

The datasets generated and analyzed during the current study are available from the corresponding author on reasonable request.
